# Modulation of Heart Rate Variability following PAP Ion Magnetic Induction Intervention in Subjects with Chronic Musculoskeletal Pain: A Pilot Randomized Controlled Study

**DOI:** 10.3390/ijerph20053934

**Published:** 2023-02-22

**Authors:** Antonio Viti, Giulia Panconi, Sara Guarducci, Susanna Garfagnini, Mosè Mondonico, Riccardo Bravi, Diego Minciacchi

**Affiliations:** 1 Centro Fisioterapico Apuano, Via delle Contrade 242, 55047 Lucca, Italy; 2 Department of Experimental and Clinical Medicine, University of Florence, Largo Brambilla 3, 50134 Florence, Italy

**Keywords:** heart rate variability, PAPIMI, autonomic nervous system, parasympathetic effect, chronic musculoskeletal pain

## Abstract

Heart rate variability (HRV) analysis has emerged as a simple and non-invasive technique to indirectly evaluate the autonomic nervous system (ANS), and it is considered a sensible and advanced index of health status. Pulsed electromagnetic fields (PEMFs) are widely used in clinical settings for improving the health status of individuals with chronic musculoskeletal pain. The aim of the present single-blind, randomized, placebo-controlled parallel pilot study was to investigate the acute effect of a single session of PEMFs stimulation by a PAP ion magnetic induction (PAPIMI) device on ANS activity, as measured by HRV, in patients with chronic musculoskeletal pain, and compare such effect with that induced by a sham (control) PAPIMI inductor. Thirty-two patients were randomized into two groups: PAPIMI intervention (PAP) (n = 17) and sham PAPIMI intervention (SHAM-PAP) (n = 15). HRV was assessed before and following the interventions. The PAP group showed a significant increase in all values of the time-domain parameters (SDNN, RMSSD, NN50, and pNN50) and the HF component of HRV, suggesting a parasympathetic effect. In contrast, the SHAM-PAP group showed no significant differences in all HRV indices following the intervention. Preliminary findings suggested that PAPIMI inductor could influence ANS activity and provided initial evidence of the potential physiological response induced by the PAPIMI device.

## 1. Introduction

The autonomic nervous system (ANS) plays a critical role in the regulation and coordination of the physiological processes in human biological organisms, and individual differences in autonomic balance have long been associated with health and pathological conditions [1,2,3].

The ANS is assumed to be composed of two major antagonistic subsystems that commonly act in dynamic balance: the sympathetic branch, involved in regulating energy mobilization, and the parasympathetic branch, implicated in vegetative and restorative functions [4,5]. Both branches innervate the intrinsic cardiac nervous system and project to sinoatrial and atrioventricular nodes, together with selected cardiac muscle regions, with the sympathetic system implicated in increasing the heart rate (HR) through the activity of efferent sympathetic nerves and the parasympathetic system responsible for slowing the HR by acting via vagal nerves [4].This dynamic relationship between the sympathetic and parasympathetic systems provides greater flexibility and favors adaptations of the organism in accommodating rapidly changing physiological and environmental demands [2]. This is in accordance with the complexity theory claiming that patterns of organized variability generated by a dynamic relationship among system elements, rather than static levels, preserve stability, adaptability, and health [1,2,6,7,8].

The dynamic balance between distinct components of the ANS can be threatened and altered by metabolic (inner) and environmental (external) stimuli, which may cause a condition of autonomic imbalance. Specifically, the loss of autonomic balance, in which one branch of the ANS prevails over the other, is characterized by a lack of dynamic adaptability and of the health of the organism. A substantial amount of empirical evidence indicates that a host of pathological conditions and diseases are associated with autonomic dysregulation, in which, typically, the sympathetic system is hyperactive and the parasympathetic system is hypoactive [2,7].

Among the methods adopted to assess ANS function, heart rate variability (HRV) analysis has emerged as a simple and noninvasive technique to indirectly evaluate the autonomic activity, representing one of the most important quantitative markers of ANS balance/imbalance [9,10,11,12]. HRV (i.e., the oscillation in the interval between consecutive heart beats around the mean heart rate) is directly influenced by ANS activity, as the balance between sympathetic excitation and vagal inhibition of sinoatrial node regulates HR [4,9,10,11,12,13,14]. Therefore, HRV can be identified as reflecting ANS function and used to reveal functional changes in sympathovagal balance. In addition, changes in HRV patterns can provide a sensible and advanced index of the health status of the individual. Indeed, while high values of HRV are reported as an indicator of good adaptation and associated with efficient autonomic mechanisms in healthy individuals, low values of HRV are considered an indicator of abnormal and insufficient adaptation of the ANS, which is related to poor physiological function in individuals [14,15,16,17].

As regards the link to pathological conditions, it was reported that ANS imbalance is associated with chronic musculoskeletal pain. This common physiological disfunction is defined as a pain that affects bones, muscles, ligaments, tendons, and even nerves; it comprises a number of different pain syndromes such as chronic neck pain, shoulder pain, low back pain, and also pain associated with osteoarthritis and rheumatoid arthritis [18,19]. As shown in previous studies, there is a strict connection between systems involved in autonomic control and those involved in pain perception [20,21]. For instance, alterations in ANS control influence the central processing and subjective experience of pain [22], with a lower activity of the parasympathetic branch, which may place a person at greater risk of chronic pain due to the diminished capacity to respond adaptively to threats (i.e., pain). Consistently, individuals living in a state of chronic pain revealed a decreased HRV, implicating parasympathetic nervous system dysregulation [23]. Reduced HRV, as an implication of ANS imbalance, was reported in subjects with chronic shoulder pain [24] and low back pain [25] as compared to healthy controls. In a more recent study, the most intense and disabling neck pain was shown to be correlated with the worst HRV indices [26]. Thus, HRV seems to represent a valuable tool for assessing changes in pain status in patients suffering from musculoskeletal disorders and was recently suggested as a more reliable method, compared to patient-reported subjective pain scales, of detecting the effectiveness of a treatment [27].

Different non-pharmacologic approaches are available for relieving the pain associated with musculoskeletal disorders [28]. Among them, one approach that was shown in clinical use to be effectively improving the health status of individuals with musculoskeletal pain is pulsed electromagnetic fields (PEMFs). Several studies have reported that exposure to specific PEMF frequencies can be effective in reducing pain intensity and improving functionality in a variety of chronic pain populations, such as patients with low back pain [29,30,31], patellofemoral pain syndrome [32], shoulder impingement syndrome [33], and lateral epicondylitis [34]. Additionally, PEMFs modulate inflammation in injured tissues; this results in enhanced functional recovery. Hence, it is a potential novel nonpharmaceutical means for the treatment of pain in musculoskeletal disorders [35]. However, although PEMFs were widely used in clinical settings for the treatment of musculoskeletal pain and the exposure to electromagnetic fields was also found to enhance parasympathetic activity [36,37,38,39], no studies investigated the effects of PEMFs on HRV in patients with musculoskeletal pain. Since HRV is considered a valuable tool to evaluate health status in individuals with chronic pain disorders, there is a necessity to study the effects of PEMFs in modulating HRV in these populations.

The PAP ion magnetic induction (PAPIMI) device is a certified and approved medical instrument that belongs to the large family of PEMFs. This device provides a non-invasive, painless, contact-free ion-induction therapy that was shown to be effective for the treatment of refractory seizures [40], skin wound healing [41], and fatigue syndrome symptoms [42]. Although PEMFs are also used as a treatment for pain disorders in clinical use, no studies are present in the literature regarding the effect of PAPIMI inductor in modulating HRV in subjects with musculoskeletal pain.

Therefore, the aim of the present single-blind, randomized, placebo-controlled parallel pilot study was to investigate the acute effect of a single-session treatment of PEMFs stimulation by PAPIMI inductor on ANS balance, as measured by HRV, in patients suffering from chronic musculoskeletal pain, and compare such effect with that induced by a sham (control) PAPIMI inductor. We hypothesized that PAPIMI intervention, as compared to the sham one, could induce a more effective parasympathetic response, which would result in an increased HRV.

## 2. Materials and Methods

### 2.1. Participants

From a population of 95 patients, 32 subjects (17 men and 15 women) with painful symptoms in the musculoskeletal system, aged between 28 and 68 years old (50.06 ± 10.85), were enrolled in the study. Patients were recruited from April to May 2022 from the physiotherapy center “Centro FisioterapicoApuano” in Lucca (Italy) where the study was also conducted.

All patients who were considered eligible for the study had chronic musculoskeletal pain (of more than 3 months’ duration) [43] and had not received any PAPIMI treatment before our investigation. Exclusion criteria were intended to avoid conditions that might have significant autonomic effect and included any cardiovascular, neurological and schizoaffective disorders, current pregnancy or breastfeeding, and menstrual flow during the session [30,44,45]. Smokers, as well as participants involved in drug abuse, were excluded [44,46]. Other exclusion criteria were the use of an anticoagulation therapy and the presence in the body of ring-shaped metals or any electronically based implanted devices such as pacemakers, defibrillators, spinal cord stimulators, insulin pumps, and cochlear implants [30]. Moreover, participants were asked to refrain from alcohol, caffeine and cardiovascular exercise for 24 h prior to the experimental session.

Participants were recruited after being evaluated by a physiotherapist from the center, who confirmed a diagnosis of musculoskeletal pain syndrome and collected participants’ pharmacological information through an in-person interview. Prior to the start of the study, informed consent was read and signed by each patient. The study was approved by the local institutional review committee (Prot. N. 12774E_spe). Each subject was blind to study design, outcome, and group allocation.

### 2.2. Experimental Procedure

**Figure 1:**
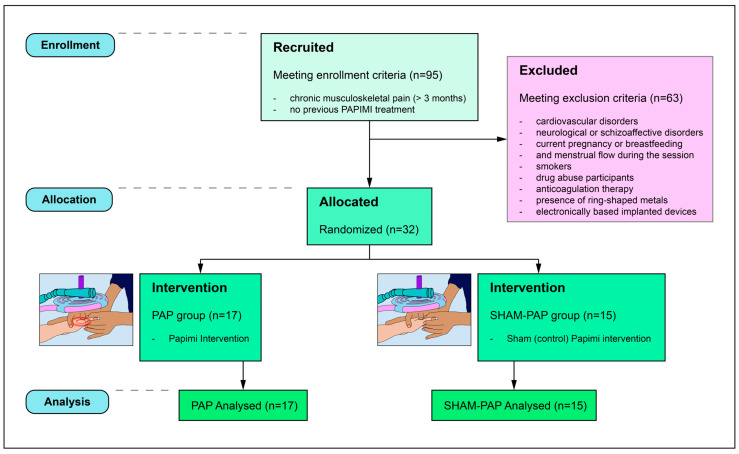
Flow-chart of the study.

Patients were randomized into two different groups: PAPIMI intervention (PAP) (n = 17) and sham (control) PAPIMI intervention (SHAM-PAP) (n = 15) (Figure 1). Each patient received a single-session treatment based on the allocation group. Each intervention was preceded and immediately followed by the HRV measurement. Information about true and sham interventions was given to patients at the end of the experimental session. The entire procedure lasted approximately 50 min for each patient. Treatments and evaluations were executed in the same room, with stable temperature and humidity, to avoid any influence on ANS activity [44,47].

### 2.3. Interventions

PAP and SHAM-PAP interventions were administered to all patients by a single licensed and registered physiotherapist.

#### 2.3.1. PAPIMI Intervention

**Figure 2:**
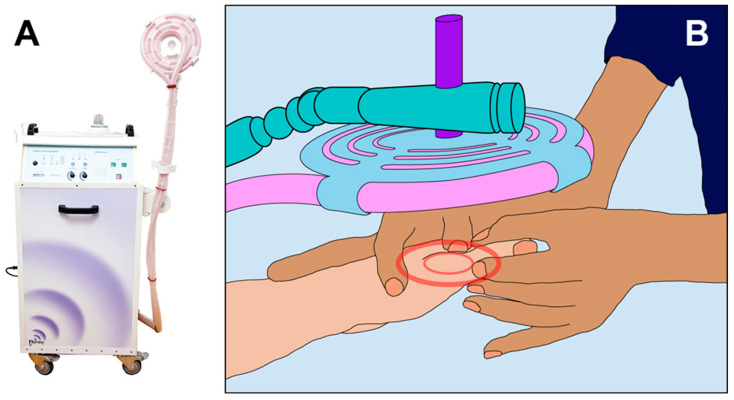
PAPIMI intervention. (**A**) PAPIMI^TM^ device. (**B**) The physiotherapist applied the treatment coil over the referred pain zone and then adjusted the distance between the coil and the skin surface of the patient.

Patients allocated to the PAP intervention group were treated with a PAP ion magnetic inductor (PAPIMI^TM^, Pulse Dynamics, Athens, Greece. Model ASKLIPIOS. Manufacturer characteristics: Input 230 V-50/60 Hz; Power 2500 VA (1.5 KW); Fuse 10 Amp, slow) [48] (Figure 2).

The PAPIMI is an approved medical device that belongs to the large family of PEMFs, and it is based on the principle of ion induction. The device is composed of a capacitor that stores electrical energy, which is then conducted through a plasma chamber (spark gap). The plasma chamber technology is used to discharge a very high voltage from the capacitor into a spiral coil applicator to create high intensity electromagnetic pulses.

Due to very high voltages reaching up to 40kV and peak currents of several 10 kA, an energy output per pulse of about 60 W (joules) with a magnetic induction of 50–150 mT is achieved. Each pulse has a base frequency of about 240 kHz and a duration of about 50 μs. Additionally, the pulse repetition rate can be varied between 1 and 8 Hz. The generated pulses are transmitted without any direct contact with the body, which is penetrated by the induction field. This latter generates weak electric currents having the same intensity and frequency as the originally generated magnetic pulse [48,49].

The PAPIMI intervention was tailored according to the individual’s needs and administered through the spiral coil applicator. The treatment coil used for the intervention (diameter 18 cm) enhanced ion induction to 100 mT and was centered over the referred pain zone. The applicator was held at a distance of approximately 4 cm from the relevant part of the body (Figure 2). The PAPIMI treatment never exceeded 30 min of application, in accordance with the manufacturer’s recommendations [48].

#### 2.3.2. Sham (Control) Papimi Intervention

Patients allocated to the sham intervention group received an inactive PEMF treatment and underwent the same procedure as that of the experimental group. The PAPIMI sham device was externally identical to the active one, except that it was deactivated prior to the exposure so that no pulsed electromagnetic fields were generated.

### 2.4. Data Collection Measurements

Before and immediately after each intervention, HRV was evaluated. Patients were recorded for a short period of 5 min in a resting state using a Polar H7 heart belt (Polar Electro, Kempele, Finland) [9]. During the recording session, subjects were instructed to sit comfortably as still as possible, and to perform spontaneous breathing without talking in order not to contaminate the record of HRV.

R-R intervals were subsequently evaluated using Kubios HRV Premium 3.4.1 desktop software (Kubios OY, Kuopio, Finland) to calculate all the HRV parameters considered in this study (see Section 2.5). R-R series of 300 cardiac beats were automatically analyzed by the software, which corrected the outliers and artifacts. If fewer than 300 R-R values were recorded, all of them were considered for the analysis. Conversely, if more than 300 R-R values were recorded, a sequence of 300 beats was selected randomly inside the period of analysis [44].

### 2.5. Outcome Measures

Pre- and post-treatment values of HRV were examined. HRV evaluation, based on processing R-R intervals, was divided into time- and frequency-domain linear analysis [50].

The following time-domain parameters of HRV were considered: standard deviation of adjacent NN intervals (SDNN), the root mean square of successive R-R interval differences (RMSSD), the number of adjacent NN intervals differing by more than 50 milliseconds (NN50), and the percentage of successive R-R intervals differing by more than 50 ms (pNN50). RMSSD, NN50, and pNN50 are correlated with parasympathetic activation [4,51,52], while SDNN is influenced by both components of the ANS [51], even though in short-term resting recordings the main source of the variation is parasympathetically mediated respiratory sinus arrhythmia [4,52]. An increase in SDNN, RMSSD, NN50, and pNN50 values indicates a parasympathetic activation [52].

The frequency-domain index of HRV analyzed in this study was the high frequency (HF) band, from 0.15 to 0.4 Hz. The HF band is indicative of parasympathetic activity and is called the respiratory band because it corresponds to the HR variations related to the respiratory cycle [5,9,52,53]. High values of HF indicate a parasympathetic predominance [52]. We did not include the very low frequency band, the low frequency band, and the LF/HF ratio, as their physiological interpretation has been considered unclear [9,54,55,56,57].

### 2.6. Statistical Analysis

All the subjects’ demographic data as well as their HRV parameters were described using arithmetic means, standard deviations (SDs), and standard errors of the mean (SEM). The chi-squared test was used to test for gender differences between groups, and results were expressed in percentages. In addition, group differences were evaluated for age, body mass index (BMI) and HR using the two-sample *t*-test with INTERVENTION GROUP as the between-subject factor (two levels: PAP and SHAM-PAP).

In the present study, HRV analysis was executed using the restricted weak stationarity (RWS) test proposed by Porta and colleagues (2004), which assesses stationarity (i.e., the steadiness of the mean and variance, respectively) over M patterns [13]. This was necessary due to the fact that the presence of non-stationarities in short HRV recordings was shown to affect HRV data [13,58]. First, the Kolmogorov–Smirnov goodness-of-fit test was applied to test the normality of R-R distribution (*p* < 0.05). In cases of non-normal distribution, a log transformation was utilized and normality retested to justify the use of parametric tests. If data were still non-normally distributed, then the R-R series were finally rejected [13]. Following this, M patterns were randomly selected from a set of sequences of length L, and a test for normality was performed [13]. The stationarity of the variance over M patterns was checked by performing a Bartlett test if a normal distribution of data was confirmed, otherwise Levene’s test using the median was applied. Then, the stationarity of mean over M patterns was evaluated via an analysis of variance (ANOVA) if the null hypothesis of a normal distribution of data could not be rejected, alternatively a Kruskal–Wallis test was adopted.

To assess the effect of PAP and SHAM-PAP interventions on the ANS, each time-domain parameter of HRV (SDNN, RMSSD, NN50, and pNN50, respectively) and the frequency-domain index of HRV (HF band) taken into account for this study were entered in a mixed analysis of variance (ANOVA) with INTERVENTION GROUP as the between-subject factor (two levels: PAP and SHAM-PAP) and TIME as a within-subject factor (two levels: pre-intervention and post-intervention). Post hoc pairwise analysis was conducted with Bonferroni adjustment for multiple comparisons. The threshold for statistical significance was set to *p* < 0.05. Effect sizes were calculated as either partial eta squared (*η*^2^*_p_*) or Cohen’s *d* based on the analysis. All data analysis was conducted using IBM’s SPSS software version 28.

## 3. Results

### 3.1. Sample Characteristics and HRV Pre-Intervention Scores

Out of 95 patients, 32 were included in this study and randomized into two groups (PAP and SHAM-PAP). Patients who experienced any adverse effect from treatment would have been excluded from the study. Since no adverse events were reported, none of the participants was lost for this reason, and all received the treatment (Figure 1). Each participant recorded no fewer than 300 cardiac beats.

The demographics, characteristics, and baseline values of HRV parameters are presented in Table 1, showing no statistically significant differences between the PAP and SHAM-PAP groups.

### 3.2. HRV Post-Intervention Scores

**Figure 3:**
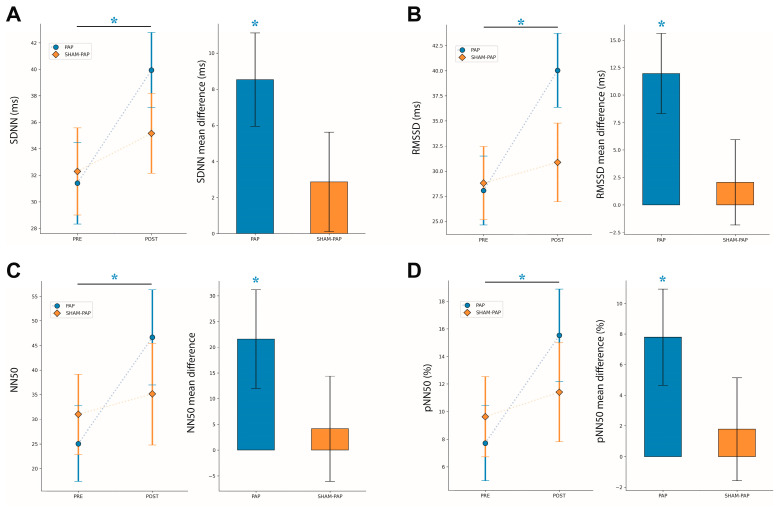
Time-domain parameters of HRV at rest, pre- and post-treatment. (**A**) SDNN, (**B**) RMSSD, (**C**) NN50, (**D**) pNN50. Data presented are mean ± standard errors of the mean (SEM). Asterisk marks a significant difference (*p* < 0.05).

**Figure 4:**
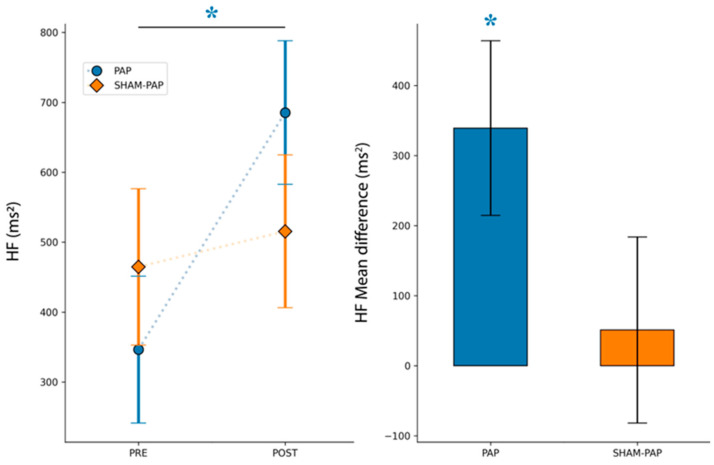
Frequency-domain parameter of HRV at rest, pre- and post-treatment. HF band. Data presented are mean ± standard errors of the mean (SEM). Asterisk marks a significant difference (*p* < 0.05).

The analyzed parameters of time- and frequency-domain of HRV after PAP and SHAM-PAP interventions are reported in Figure 3 and Figure 4, respectively.

#### 3.2.1. SDNN

For the SDNN index, the results of the mixed ANOVA showed a significant main effect for TIME [*F*_(1,30)_ = 9.105, *p* < 0.005, *η*^2^*_p_* = 0.233]. In contrast, no significant main effect was found for INTERVENTION [*F*_(1,30)_ = 0.241, *p* = 0.627, *η*^2^*_p_* = 0.008] and interaction TIME by INTERVENTION [*F*_(1,30)_ = 2.248, *p* = 0.144, *η*^2^*_p_* = 0.070]. Post hoc pairwise comparisons showed a significant increase (pre–post interventions) in SDNN scores in PAP group [mean difference_(post-pre)_ = 8.53 (SEM = 2.59), *p* = 0.003, *d* = 0.651] but not in SHAM-PAP group [mean difference_(post-pre)_ = 2.87 (SEM = 2.75), *p* = 0.306].

#### 3.2.2. RMSSD

For the RMSSD index, a significant main effect for TIME [*F*_(1,30)_ = 6.971, *p* = 0.013, *η*^2^*_p_* = 0.189] was revealed, while no significant main effect for INTERVENTION [*F*_(1,30)_ = 0.865, *p* = 0.360, *η*^2^*_p_* = 0.028] and interaction TIME by INTERVENTION [*F*_(1,30)_ = 3.471, *p* = 0.072, *η*^2^*_p_* = 0.104] was shown. Post hoc pairwise comparisons revealed a significant increase (pre–post interventions) in RMSSD scores in PAP group [mean difference_(post-pre)_ = 11.98 (SEM = 3.64), *p* = 0.003, *d* = 0.700] but not in SHAM-PAP group [mean difference_(post-pre)_ = 2.07 (SEM = 3.88), *p* = 0.598].

#### 3.2.3. NN50

For the NN50 index, no significant main effect for TIME [*F*_(1,30)_ = 3.364, *p* = 0.077, *η*^2^*_p_* = 0.101], INTERVENTION [*F*_(1,30)_ = 0.069, *p* = 0.795, *η*^2^*_p_* = 0.002] and interaction TIME by INTERVENTION [*F*_(1,30)_ = 1.549, *p* = 0.223, *η*^2^*_p_* = 0.049] was observed. Post hoc analysis showed a significant increase in NN50 scores following PAP intervention [mean difference_(post-pre)_ = 21.59 (SEM = 9.60), *p* = 0.032, *d* = 0.572] but not after SHAM-PAP one [mean difference_(post-pre)_ = 4.13 (SEM = 10.22), *p* = 0.689].

#### 3.2.4. pNN50

For the pNN50 index, a significant main effect for TIME [*F*_(1,30)_ = 4.345, *p* = 0.046, *η*^2^*_p_* = 0.127] was found, while no significant main effect was reported for INTERVENTION [*F*_(1,30)_ = 0.085, *p* = 0.773, *η*^2^*_p_* = 0.003]. In addition, no significant interaction TIME by INTERVENTION [*F*_(1,30)_ = 1.719, *p* = 0.200, *η*^2^*_p_* = 0.054] was shown. Post hoc analysis showed a significant increase in pNN50 scores following PAP intervention [mean difference_(post-pre)_ = 7.81 (SEM = 3.15), *p* = 0.019, *d* = 0.575] but not after SHAM-PAP one [mean difference_(post-pre)_ = 1.78 (SEM = 3.36), *p* = 0.600].

#### 3.2.5. HF

For the HF band, mixed ANOVA showed a significant main effect of TIME [*F*_(1,30)_ = 4.594, *p* = 0.040, *η*^2^*_p_* = 0.133] but no significant main effect for INTERVENTION [*F*_(1,30)_ = 0.045, *p* = 0.834, *η*^2^*_p_* = 0.001] and interaction TIME by INTERVENTION [*F*_(1,30)_ = 2.505, *p* = 0.124, *η*^2^*_p_* = 0.077]. Post hoc analysis showed a significant increase in HF component after PAP intervention [mean difference_(post-pre)_ = 339.06 (SEM = 124.60), *p* = 0.011, *d* = 0.830] but not after SHAM-PAP one [mean difference_(post-pre)_ = 51.00 (SEM = 132.65), *p* = 0.703].

## 4. Discussion

In the current study, we investigated the influence of a single session of PEMFs stimulation using PAPIMI device on modulating ANS activity in patients with chronic musculoskeletal pain. An HRV analysis was employed in order to detect changes in vagal tone after the intervention.

Our results showed that all values of time-domain parameters and HF component of HRV significantly increased following PAP treatment, indicating that PAPIMI device modulated ANS activity by inducing a parasympathetic response. In contrast, no significant differences in all HRV indices were found following the SHAM-PAP intervention.

In clinical practice, PEMFs are widely used as a safe and non-pharmacologic intervention for the treatment of musculoskeletal disorders [59]. The benefits of this approach were evidenced by previous reports showing an improvement in health status after PEMFs treatment in a variety of populations suffering from chronic pain [29,30,31,32,33,34].

Among PEMFs, PAPIMI inductor represents a promising ion magnetic field device which has proven to help restore health status in subjects with pathological conditions [40,42] characterized by reduced parasympathetic tone [60,61]. However, so far, no study has investigated the effects of PAPIMI device on modulating ANS activity.

Our findings shed light on the effectiveness of PAPIMI device in inducing a health status-related parasympathetic response, as indirectly shown by an increased HRV, in patients with chronic musculoskeletal pain. A possible hypothesis that could explain such effect is related to the influence PEMFs have on the membrane potential of body cells [40,62,63]. In this regard, an impaired blood-flow regulation in the affected tissue in patients with chronic pain was reported [64,65]. This degradation in microcirculation is speculated to reduce the level of oxygen and alter natural membrane potential (approximately −73 mV), which is crucial for transport regulation and signal transmission in healthy cells [66,67]. PEMFs, when applied on the affected area, were shown to induce cells to generate weak microelectrical currents that in turn can influence ions’ concentration, distribution, and flux, which are known to determine and establish the cell membrane potential [40,62,63,67]. Therefore, the exposure of the injured area to PAPIMI inductor might enhance the regulation in the transport of ions across the plasmatic membrane, thus contributing to raising cells to their healthy voltage potential. Specifically, the interaction between the pulsed field generated by PAPIMI inductor and the body area of application could have induced at the cell membrane level a cascade of healing events leading to the short-term restoration of a healthy state in the affected area. This re-establishment of health could be reflected in an augmented activity of the parasympathetic branch of the ANS [9,23,68,69,70], as shown by the increase in all the time-domain parameters as well as the HF band after the intervention. In this sense, our hypothesis could explain the effect of PAPIMI inductor we detected at the heart level via HRV analysis, despite the device being applied on pain-related sites of the body. However, our interpretation should be taken with caution and calls for additional future studies.

Finally, this is the first study investigating the influence of PAPIMI device on the ANS activity in patients with chronic musculoskeletal pain. Taking into account the low risk of side effects and the high tolerance of PEMFs intervention, findings from our pilot study indicate that PAPIMI inductor could be considered a potentially promising device in clinical practice for the treatment and management of chronic musculoskeletal pain, thus further contributing to improvements in patient health status. To the best of our knowledge, in the literature, only two studies have explored the effects of PAPIMI inductor in human subjects [40,42]. They used a single-subject research design and considered other pathological conditions, such as refractory seizures [40] and fatigue syndrome symptoms [42]. Moreover, these studies did not employ an HRV analysis to quantitatively evaluate the effectiveness of PAPIMI treatment. Therefore, sample characteristics and methodological differences between our study and previous reports make it difficult to compare the results.

Some limitations concerning the present pilot study should be addressed. First, the number of participants remains relatively small. Second, a comparison with a control group of healthy subjects was not planned for this study. Future non-pilot studies should preferably include a larger patient sample and a group of healthy individuals as well. Third, we did not provide an evaluation of subjects’ feeling of well-being or pain perception that could provide information on the responsiveness of patients to PAPIMI intervention. Nevertheless, it is worth mentioning that the analysis of the cardiovascular autonomic control seems to be more sensitive than a self-reported measure of pain for estimating the effectiveness of a non-pharmacological treatment in patients with musculoskeletal pain [27]. Fourth, another limitation to consider is related to the employment of HF component as a parasympathetic activity predictor. Although a strong correlation was revealed between HF power and cardiac parasympathetic activity [2,71,72,73,74,75], HF component may also be partially modulated by sympathetic nerve activation [74,76,77]. Finally, there is a need to be aware of the possible misrecognition mistakes of R-R intervals made by HRV software tools that could influence the accuracy of HRV analysis, specifically, that related to time-domain parameters (e.g., SDNN) [78]. Therefore, future investigations employing a combination of measures, including skin conductance in addition to HRV analysis, could provide further insight into the effect of PAPIMI inductor on ANS activity [52].

Future studies exploring the effect of PAP treatment in subjects with chronic musculoskeletal pain should be carried out with a longer intervention period [30,31,32,33]. Moreover, comparing the magnitude of the effect of PAPIMI inductor with that induced by other PEMFs devices could be of value. Furthermore, since it is globally recognized that there is a necessity for a plurality of interventions in order to obtain a benefit for chronic musculoskeletal pain [79], it might be of interest to evaluate the additive effect of PAPIMI intervention combined with other non-pharmacological treatments (e.g., physical exercise) [32,33]. Finally, in the last years, the relationship between mood state and chronic musculoskeletal pain has been largely investigated in human and animal models, with several studies indicating depression and anxiety as consequences of protracted pain [80,81,82,83,84,85,86,87,88,89,90]. Therefore, future explorations could investigate the effects of PAPIMI inductor on reducing the level of depression and anxiety as the function of changes in cardiovascular autonomic control.

## 5. Conclusions

In this study we investigated the effect of PAPIMI intervention on ANS activity in patients with chronic musculoskeletal pain. Results showed that PAPIMI inductor, as compared to a sham (control), induced a significant effective parasympathetic response, which resulted in an increased HRV. Since musculoskeletal pain conditions have been associated with a reduced HRV [23,24,25,26], such acute autonomic change observed following PAPIMI intervention could be interpreted as a health status-related parasympathetic response. This is the first time the association between PAPIMI inductor and autonomic activity has been quantified via an HRV analysis and has provided initial evidence on the potential physiological response induced by PAPIMI inductor.

## Figures and Tables

**Table 1:** Baseline values of PAPIMI (PAP) and Sham PAPIMI (SHAM-PAP) groups.

	PAP	SHAM-PAP	*p* Value
Age (years) ^a,§^	49.18 ± 13.02	51.07 ± 9.42	0.63
BMI (kg/m^2^) ^a,§^	24.52 ± 3.62	25.19 ± 3.60	0.58
Male ^b,$^	10 (58.82)	7 (46.67)	0.47
HR (bpm) ^a,§^	72.18 ± 11.00	69.17 ± 6.83	0.37
SDNN (ms) ^a,§^	31.41 ± 13.74	32.30 ± 11.44	0.85
RMSSD (ms) ^a,§^	28.07 ± 16.67	28.80 ± 10.50	0.88
NN50 ^a,§^	25.06 ± 32.92	31.01 ± 29.86	0.60
pNN50 (%) ^a,§^	7.72 ± 11.75	9.63 ± 10.60	0.64
HF (ms^2^) ^a,§^	346.35 ± 458.01	464.67 ± 402.16	0.45

Numbers in table are mean ± standard deviations (SDs) ^a^, n (%) ^b^, two sample *t*-test ^§^, and chi square ^$^.

## Data Availability

The data presented in this study are available on request from the corresponding author. The data are not publicly available due to privacy issues.
